# Fluoren-9-one oxime

**DOI:** 10.1107/S1600536814002669

**Published:** 2014-02-08

**Authors:** Bernhard Bugenhagen, Yosef Al Jasem, Mariam Al-Azani, Thies Thiemann

**Affiliations:** a Institute for Inorganic and Applied Chemistry, University of Hamburg, Martin-Luther-King-Platz 6, D-20146 Hamburg, Germany; b Department of Chemical Engineering, United Arab Emirates University, AL Ain, Abu Dhabi, United Arab Emirates; c Department of Chemistry, United Arab Emirates University, AL Ain, Abu Dhabi, United Arab Emirates

## Abstract

In the title mol­ecule, C_13_H_9_NO, the fluorene system and the oxime group non-H atoms are essentially coplanar, with a maximum deviation from the fluorene mean plane of 0.079 (2) Å for the oxime O atom. A short intra­molecular C—H⋯O generates an *S*(6) ring. In the crystal, mol­ecules related by a twofold screw axis are connected by O—H⋯N hydrogen bonds, forming [100] chains Within these chains, mol­ecules related by a unit translation along [100] show π–π stacking inter­actions between their fluorene ring systems with an inter­planar distance of 3.347 (2) Å. The dihedral angle between the fluorene units of adjacent mol­ecules along the helix is 88.40 (2)°. There is a short C—H⋯π contact between the fluorene groups belonging to neighbouring chains.

## Related literature   

For the original procedure for the preparation of the title compound, see: Moore & Huntress (1927 ▶). For the use of the title compound as a starting material for the synthesis of bioactive compounds, see: Amlaiky *et al.* (1983 ▶); Ni *et al.* (2009 ▶); Rad *et al.* (2012 ▶).
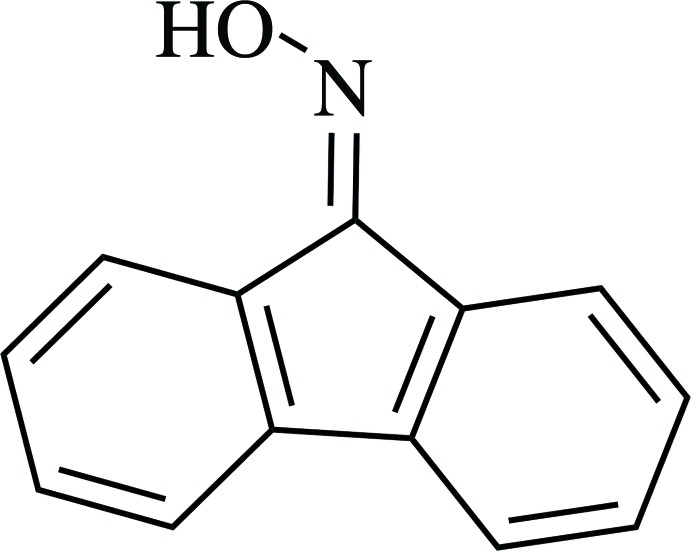


## Experimental   

### 

#### Crystal data  

C_13_H_9_NO
*M* *_r_* = 195.21
Orthorhombic, 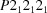

*a* = 4.8009 (1) Å
*b* = 12.2309 (2) Å
*c* = 16.0247 (3) Å
*V* = 940.96 (3) Å^3^
*Z* = 4
Cu *K*α radiation
μ = 0.70 mm^−1^
*T* = 100 K
0.16 × 0.13 × 0.13 mm

#### Data collection  

Agilent SuperNova (Dual, Cu at zero, Atlas) diffractometer
Absorption correction: multi-scan (*CrysAlis PRO*; Agilent, 2013 ▶) *T*_min_ = 0.890, *T*_max_ = 1.000
7588 measured reflections
1942 independent reflections
1865 reflections with *I* > 2σ(*I*)
*R*_int_ = 0.029

#### Refinement  

*R*[*F* ^2^ > 2σ(*F* ^2^)] = 0.029
*wR*(*F* ^2^) = 0.075
*S* = 1.05
1942 reflections
140 parameters
H atoms treated by a mixture of independent and constrained refinement
Δρ_max_ = 0.13 e Å^−3^
Δρ_min_ = −0.18 e Å^−3^
Absolute structure: Flack parameter determined using 735 quotients [(*I* ^+^)−(*I* ^−^)]/[(*I* ^+^)+(*I* ^−^)] (Parsons *et al.*, 2013 ▶)
Absolute structure parameter: 0.16 (13)

### 

Data collection: *CrysAlis PRO* (Agilent, 2013 ▶); cell refinement: *CrysAlis PRO*; data reduction: *CrysAlis PRO*; program(s) used to solve structure: *SHELXS97* (Sheldrick, 2008 ▶); program(s) used to refine structure: *SHELXL97* (Sheldrick, 2008 ▶) within *OLEX2* (Dolomanov *et al.*, 2009 ▶); molecular graphics: *PLATON* (Spek, 2009 ▶) and *Mercury* (Macrae *et al.*, 2008 ▶); software used to prepare material for publication: *SHELXL97* and *PLATON*.

## Supplementary Material

Crystal structure: contains datablock(s) I. DOI: 10.1107/S1600536814002669/gk2601sup1.cif

Structure factors: contains datablock(s) I. DOI: 10.1107/S1600536814002669/gk2601Isup2.hkl

Supporting information file. DOI: 10.1107/S1600536814002669/gk2601Isup3.cml

CCDC reference:

Additional supporting information:  crystallographic information; 3D view; checkCIF report

## Figures and Tables

**Table 1:** Hydrogen-bond geometry (Å, °) *Cg*1 is the centroid of the C2–C7 ring.

*D*—H⋯*A*	*D*—H	H⋯*A*	*D*⋯*A*	*D*—H⋯*A*
C12—H12⋯O1	0.95	2.38	2.898 (2)	114
O1—H1⋯N1^i^	0.98 (3)	1.80 (3)	2.7758 (18)	169 (3)
C5—H5⋯*Cg*1^ii^	0.95	3.08	3.873	142

Symmetry codes: (i) 
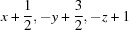
; (ii) 
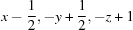
.

**Fig. 1.**
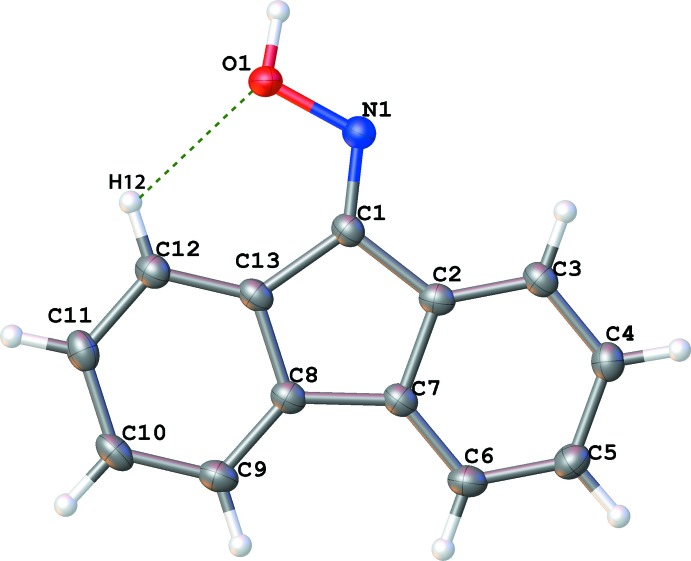
A view of the title molecule with displacement ellipsoids shown at the 50% probability level, showing the intramolecular contact within the molecule.

**Fig. 2.**
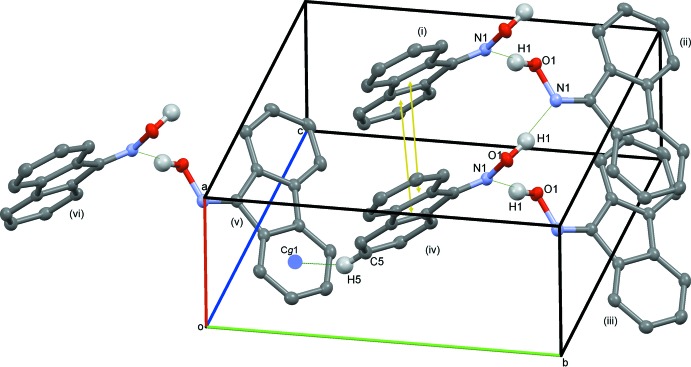
Intermolecular interactions between molecules of the title compound, including the π–π stacking interactions (represented by arrows in yellow). [Symmetry codes: i: 1 + x,y,z; ii: 1/2 + x,1.5 - y,1 - z; iii: -1/2 + x,1.5 - y,1 - z; iv: x, y, z; v: -1/2 + x, 1/2 - y, 1 - z; vi: x, -1 + y, z]

**Fig. 3.**
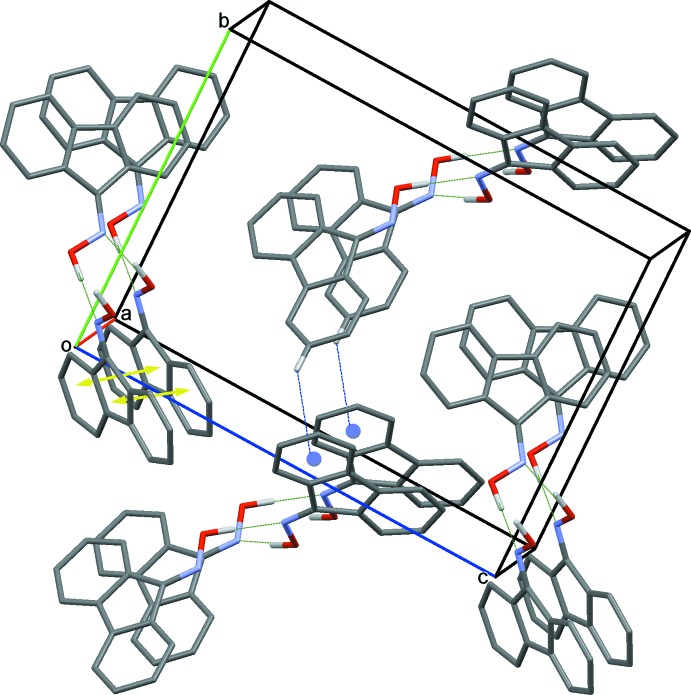
The crystal packing diagram showing the O—H···N close contacts (colored in green) between adjacent molecules in each helical bonding network, C—H···π intermolecular interaction between molecules within different helices (colored in blue) and π–π stacking interactions (arrows in yellow).

## supplementary crystallographic information

### 1. Comment

The title compound, which was prepared according to a known procedure (Moore & Huntress, 1927) has found extensive use as a starting material for preparation of medicinal active compounds such as novel cyclophilin A inhibitors (Ni *et al.*, 2009), novel analogs of beta-adrenoceptor antagonists (Rad *et al.*, 2012) and beta-blocker (Amlaiky *et al.*, 1983).

The ring atoms, N and O atoms in the title compound molecule, are essentially coplanar with a maximum deviation of 0.079 (2) Å for the O atom from the averaged ring plane (13 carbon atoms). The molecule in the crystal exhibits a C12—H12···O1 intramolecular interaction (Table 1), (Figure 1). The molecules related by a twofold screw axis are connected by O1—H1···N1 hydrogen bonds (Table 1) within a helical bonding network extending along the *a* axis (Figure 2). Within one helical bonding network, the neighboring molecules related by a unit translation along [100] show π–π stacking interactions between their fluorene ring systems with the interplanar distance of 3.347 (2) Å (Figure 2). The neighboring helical bonding networks in parallel alignment are linked to each other by C5—H5··· π (*Cg*1) (Table 1) close contact between their fluorene groups (Figure 3). The dihedral angle between the fluorene units of adjacent molecules along the helix is 88.40 (2)°.

### 2. Experimental

To a solution of fluoren-9-one (1.8 g, 10 mmol) in EtOH (47 ml) was given a solution of hydroxylamine hydrochloride (NH_2_OH^.^HCl, 2.75 g, 39.6 mmol) in water (7 ml), and the resulting mixture was stirred at 70 *^o^*C for 5 h. Thereafter, the reaction mixture was cooled and given into water (150 ml). The colorless precipitate was extracted with CH_2_Cl_2_ (2 × 75 ml). The organic phase was dried over anhydrous MgSO_4_ and concentrated *in vacuo* to give fluoren-9-one oxime (1.79 g, 92%) as a colorless solid, mp. 471 – 472 K; ν_max_ (KBr/cm^-1^) 3500 – 2800 (bs, OH), 1604 (w), 1602, 1450, 1405, 1317, 1156, 1089, 998, 937, 780, 732, 640; δ_H_ (400 MHz, CDCl_3_) 7.28 – 7.47 (4*H*, m), 7.62 (1*H*, d, ^3^*J* = 7.6 Hz), 7.66 (1*H*, d, ^3^*J* = 7.2 Hz), 7.77 (1*H*, d, ^3^*J* = 7.2 Hz), 8.42 (1*H*, d, ^3^*J* = 7.6 Hz); δ_C_ (100.5 MHz, CDCl_3_) 119.8 (CH), 119.9 (CH), 121.7 (CH), 128.0 (CH), 128.4 (CH), 129.7 (CH), 130.2 (CH), 130.3 (C_quat_), 131.3 (CH), 135.1 (C_quat_), 140.5 (C_quat_), 141.4 (C_quat_), 153.6 (C_quat_). Single crystals were obtained from cold CH_2_Cl_2_.

### 3. Refinement

All carbon-bound hydrogen atoms, except the H of the OH group which was freely refined, were placed in calculated positions with C—H distance of 0.95 Å and refined as riding with *Uiso*(H) = 1.2*Ueq*(C).

### Crystal data

**Table:**

C_13_H_9_NO	*D*_x_ = 1.378 Mg m^−^^3^
*M**_r_* = 195.21	Melting point = 471–472 K
Orthorhombic, *P*2_1_2_1_2_1_	Cu *K*α radiation, λ = 1.5418 Å
*a* = 4.8009 (1) Å	Cell parameters from 3850 reflections
*b* = 12.2309 (2) Å	θ = 4.5–76.1°
*c* = 16.0247 (3) Å	µ = 0.70 mm^−^^1^
*V* = 940.96 (3) Å^3^	*T* = 100 K
*Z* = 4	Block, colourless
*F*(000) = 408	0.16 × 0.13 × 0.13 mm

### Data collection

**Table:**

Agilent SuperNova (Dual, Cu at zero, Atlas) diffractometer	1942 independent reflections
Radiation source: SuperNova (Cu) X-ray Source	1865 reflections with *I* > 2σ(*I*)
Mirror monochromator	*R*_int_ = 0.029
Detector resolution: 10.4127 pixels mm^-1^	θ_max_ = 76.3°, θ_min_ = 4.6°
ω scans	*h* = −6→5
Absorption correction: multi-scan (*CrysAlis PRO*; Agilent, 2013)	*k* = −14→15
*T*_min_ = 0.890, *T*_max_ = 1.000	*l* = −19→20
7588 measured reflections	

### Refinement

**Table:**

Refinement on *F*^2^	Hydrogen site location: difference Fourier map
Least-squares matrix: full	H atoms treated by a mixture of independent and constrained refinement
*R*[*F*^2^ > 2σ(*F*^2^)] = 0.029	*w* = 1/[σ^2^(*F*_o_^2^) + (0.0384*P*)^2^ + 0.1723*P*] where *P* = (*F*_o_^2^ + 2*F*_c_^2^)/3
*wR*(*F*^2^) = 0.075	(Δ/σ)_max_ < 0.001
*S* = 1.05	Δρ_max_ = 0.13 e Å^−^^3^
1942 reflections	Δρ_min_ = −0.18 e Å^−^^3^
140 parameters	Absolute structure: Flack parameter determined using 735 quotients [(*I*^+^)-(*I*^-^)]/[(*I*^+^)+(*I*^-^)] (Parsons *et al.*, 2013)
0 restraints	Absolute structure parameter: 0.16 (13)
Primary atom site location: structure-invariant direct methods	

### Special details

**Table:**

Geometry. All e.s.d.'s (except the e.s.d. in the dihedral angle between two l.s. planes) are estimated using the full covariance matrix. The cell e.s.d.'s are taken into account individually in the estimation of e.s.d.'s in distances, angles and torsion angles; correlations between e.s.d.'s in cell parameters are only used when they are defined by crystal symmetry. An approximate (isotropic) treatment of cell e.s.d.'s is used for estimating e.s.d.'s involving l.s. planes.

### Fractional atomic coordinates and isotropic or equivalent isotropic displacement parameters (Å^2^)

**Table:**

	*x*	*y*	*z*	*U*_iso_*/*U*_eq_	
C1	0.5506 (4)	0.58330 (13)	0.40422 (10)	0.0189 (4)	
C10	0.8197 (4)	0.44386 (15)	0.17647 (10)	0.0251 (4)	
C11	0.9543 (4)	0.53953 (15)	0.20052 (10)	0.0242 (4)	
C12	0.8830 (4)	0.59306 (14)	0.27456 (10)	0.0219 (4)	
C13	0.6741 (4)	0.54803 (13)	0.32367 (10)	0.0196 (3)	
C2	0.3367 (4)	0.50126 (13)	0.42679 (10)	0.0194 (3)	
C3	0.1613 (4)	0.49391 (13)	0.49503 (10)	0.0209 (3)	
C4	−0.0178 (4)	0.40413 (13)	0.50050 (11)	0.0228 (4)	
C5	−0.0177 (4)	0.32387 (14)	0.43891 (11)	0.0246 (4)	
C6	0.1590 (4)	0.33133 (14)	0.37017 (11)	0.0235 (4)	
C7	0.3353 (4)	0.42037 (13)	0.36414 (10)	0.0195 (3)	
C8	0.5403 (4)	0.45037 (13)	0.30001 (10)	0.0198 (4)	
C9	0.6112 (4)	0.39797 (14)	0.22607 (11)	0.0239 (4)	
H1	0.898 (6)	0.773 (2)	0.4668 (17)	0.068 (9)*	
H10	0.8703	0.4093	0.1256	0.030*	
H11	1.0969	0.5689	0.1661	0.029*	
H12	0.9750	0.6584	0.2908	0.026*	
H3	0.1626	0.5485	0.5372	0.025*	
H4	−0.1408	0.3978	0.5467	0.027*	
H5	−0.1399	0.2631	0.4438	0.029*	
H6	0.1583	0.2764	0.3283	0.028*	
H9	0.5200	0.3325	0.2097	0.029*	
N1	0.6059 (3)	0.66488 (11)	0.45230 (8)	0.0202 (3)	
O1	0.8178 (3)	0.73127 (10)	0.42014 (7)	0.0233 (3)	

### Atomic displacement parameters (Å^2^)

**Table:**

	*U*^11^	*U*^22^	*U*^33^	*U*^12^	*U*^13^	*U*^23^
C1	0.0217 (9)	0.0194 (8)	0.0158 (7)	0.0029 (6)	−0.0016 (6)	0.0013 (6)
C10	0.0300 (10)	0.0290 (8)	0.0164 (7)	0.0078 (8)	−0.0003 (7)	−0.0031 (7)
C11	0.0257 (9)	0.0289 (9)	0.0179 (8)	0.0060 (7)	0.0018 (7)	0.0038 (7)
C12	0.0266 (9)	0.0206 (7)	0.0186 (8)	0.0025 (7)	−0.0013 (7)	0.0015 (6)
C13	0.0228 (9)	0.0204 (7)	0.0155 (7)	0.0050 (7)	−0.0024 (7)	−0.0007 (6)
C2	0.0216 (8)	0.0185 (7)	0.0181 (8)	0.0028 (7)	−0.0048 (7)	0.0005 (6)
C3	0.0233 (8)	0.0219 (7)	0.0174 (7)	0.0043 (7)	−0.0018 (7)	0.0005 (6)
C4	0.0223 (8)	0.0248 (8)	0.0214 (8)	0.0028 (6)	0.0007 (8)	0.0042 (7)
C5	0.0259 (9)	0.0210 (8)	0.0268 (9)	−0.0024 (7)	−0.0015 (7)	0.0030 (7)
C6	0.0277 (10)	0.0200 (7)	0.0228 (8)	0.0012 (7)	−0.0040 (7)	−0.0025 (6)
C7	0.0209 (8)	0.0200 (7)	0.0177 (7)	0.0037 (6)	−0.0021 (6)	−0.0002 (6)
C8	0.0212 (9)	0.0202 (7)	0.0181 (8)	0.0034 (7)	−0.0032 (7)	−0.0001 (6)
C9	0.0287 (10)	0.0220 (8)	0.0209 (8)	0.0030 (7)	−0.0039 (7)	−0.0034 (6)
N1	0.0236 (8)	0.0184 (6)	0.0185 (7)	0.0010 (6)	−0.0008 (6)	0.0014 (5)
O1	0.0283 (6)	0.0217 (5)	0.0198 (6)	−0.0058 (5)	0.0010 (5)	−0.0023 (4)

### Geometric parameters (Å, º)

**Table:**

C1—C13	1.484 (2)	C4—H4	0.9500
C1—C2	1.481 (2)	C5—C6	1.393 (2)
C10—C11	1.391 (3)	C5—H5	0.9500
C10—H10	0.9500	C6—C7	1.383 (2)
C11—C12	1.398 (2)	C6—H6	0.9500
C11—H11	0.9500	C7—C8	1.469 (2)
C12—C13	1.389 (2)	C8—C13	1.408 (2)
C12—H12	0.9500	C8—C9	1.389 (2)
C2—C7	1.410 (2)	C9—C10	1.396 (3)
C2—C3	1.383 (2)	C9—H9	0.9500
C3—C4	1.397 (2)	N1—C1	1.288 (2)
C3—H3	0.9500	O1—H1	0.98 (3)
C4—C5	1.392 (2)	O1—N1	1.4000 (19)
			
N1—O1—H1	107.7 (17)	C6—C7—C2	120.39 (16)
C1—N1—O1	112.25 (13)	C6—C7—C8	130.96 (16)
N1—C1—C2	121.43 (15)	C9—C8—C7	130.19 (16)
N1—C1—C13	131.53 (16)	C9—C8—C13	120.61 (16)
C2—C1—C13	107.00 (14)	C13—C8—C7	109.20 (14)
C3—C2—C1	131.26 (15)	C8—C9—H9	120.8
C3—C2—C7	120.97 (15)	C8—C9—C10	118.41 (16)
C7—C2—C1	107.75 (14)	C10—C9—H9	120.8
C2—C3—H3	120.8	C9—C10—H10	119.6
C2—C3—C4	118.38 (15)	C11—C10—C9	120.89 (16)
C4—C3—H3	120.8	C11—C10—H10	119.6
C3—C4—H4	119.7	C10—C11—H11	119.5
C5—C4—C3	120.63 (16)	C10—C11—C12	121.04 (17)
C5—C4—H4	119.7	C12—C11—H11	119.5
C4—C5—H5	119.5	C11—C12—H12	120.9
C4—C5—C6	120.97 (17)	C13—C12—C11	118.17 (17)
C6—C5—H5	119.5	C13—C12—H12	120.9
C5—C6—H6	120.7	C8—C13—C1	107.38 (15)
C7—C6—C5	118.66 (16)	C12—C13—C1	131.75 (16)
C7—C6—H6	120.7	C12—C13—C8	120.87 (15)
C2—C7—C8	108.64 (14)		
			
O1—N1—C1—C2	178.85 (14)	C5—C6—C7—C2	0.5 (3)
O1—N1—C1—C13	1.2 (2)	C5—C6—C7—C8	−179.68 (17)
N1—C1—C2—C3	1.5 (3)	C6—C7—C8—C9	1.3 (3)
N1—C1—C2—C7	−177.14 (15)	C6—C7—C8—C13	−178.35 (18)
N1—C1—C13—C8	177.82 (17)	C7—C2—C3—C4	0.0 (2)
N1—C1—C13—C12	−1.4 (3)	C7—C8—C9—C10	−178.93 (17)
C1—C2—C3—C4	−178.52 (16)	C7—C8—C13—C1	−0.88 (18)
C1—C2—C7—C6	178.38 (15)	C7—C8—C13—C12	178.46 (15)
C1—C2—C7—C8	−1.52 (18)	C8—C9—C10—C11	0.3 (3)
C2—C1—C13—C8	−0.04 (18)	C9—C8—C13—C1	179.46 (15)
C2—C1—C13—C12	−179.28 (17)	C9—C8—C13—C12	−1.2 (2)
C2—C3—C4—C5	0.4 (2)	C9—C10—C11—C12	−0.6 (3)
C2—C7—C8—C9	−178.86 (18)	C10—C11—C12—C13	0.1 (3)
C2—C7—C8—C13	1.53 (18)	C11—C12—C13—C1	179.97 (17)
C3—C2—C7—C6	−0.5 (2)	C11—C12—C13—C8	0.8 (2)
C3—C2—C7—C8	179.64 (15)	C13—C1—C2—C3	179.66 (16)
C3—C4—C5—C6	−0.4 (3)	C13—C1—C2—C7	0.98 (18)
C4—C5—C6—C7	0.0 (3)	C13—C8—C9—C10	0.6 (2)

### Hydrogen-bond geometry (Å, º)

Cg1 is the centroid of the C2–C7 ring.

**Table:**

*D*—H···*A*	*D*—H	H···*A*	*D*···*A*	*D*—H···*A*
C12—H12···O1	0.95	2.38	2.898 (2)	114
O1—H1···N1^i^	0.98 (3)	1.80 (3)	2.7758 (18)	169 (3)
C5—H5···*Cg*1^ii^	0.95	3.08	3.873	142

Symmetry codes: (i) *x*+1/2, −*y*+3/2, −*z*+1; (ii) *x*−1/2, −*y*+1/2, −*z*+1.
